# Breast cancer-derived exosomal miR-105-5p facilitates the transformation of NFs into CAFs through LATS2-NF-κB signaling: BC exosomal miR-105-5p transforms NFs into CAFs

**DOI:** 10.3724/abbs.2025017

**Published:** 2025-03-27

**Authors:** Xiaodi Ding, Zhimei Sheng, Jiayu Cui, Meimei Cui, Liying Zhang, Ruijun Feng, Yongming Wang, Wei Sun, Xiurong Zhang, Lihong Shi, Baogang Zhang

**Affiliations:** 1 Department of Pathology Shandong Second Medical University Weifang 261053 China; 2 Affiliated Hospital of Shandong Second Medical University Weifang 261041 China; 3 Department of Thoracic Surgery Translational Medical Center Weifang Second People’s Hospital (Weifang Respiratory Disease Hospital) Weifang 261041 China; 4 Department of Pharmacology Shandong Second Medical University Weifang 261053 China; 5 Department of Rehabilitation Medicine Shandong Second Medical University Weifang 261053 China

**Keywords:** breast cancer, exosomes, cancer-associated fibroblasts, tumor microenvironment, miRNAs

## Abstract

Studies of cell-to-cell activities in the tumor microenvironment (TME) have identified multiple potential targets for oncotherapy. The interplay between tumor cells and neighboring cancer-associated fibroblasts (CAFs) persists in all stages of tumor progression. In this study, we reveal that exosomes from breast cancer cells can be endocytosed into fibroblasts and transform normal fibroblasts (NFs) into CAFs and that the ability of exosomes from highly metastatic breast cancer cells is greater than that of those from poorly metastatic breast cancer cells. Further investigation reveals that exosomes from highly metastatic breast cancer cells contain much more miR-105-5p than those from poorly metastatic breast cells do and that exosomal miR-105-5p facilitates the transformation of NFs to CAFs. A detailed study reveals that RBMY1A1-dependent sorting of miR-105-5p into fibroblasts and subsequent internalization of miR-105-5p promote the transformation of NFs to CAFs by downregulating LATS2 expression and activating NF-κB signaling, which concurrently facilitates the EMT of breast cancer cells. Thus, our results indicate that exosomal miR-105-5p may be a potential target for novel therapeutic strategies to prevent the coevolution of breast cancer cells and CAFs.

## Introduction

Breast cancer is the most commonly diagnosed malignancy among women [1] and has always been one of the leading causes of cancer-related death [2]. The primary and common cause of death is typically ascribed to the continuous advancement of the tumor or the unsuccessful outcome of the implemented treatment. Numerous studies have highlighted the roles of the tumor microenvironment (TME) in breast cancer progression and the prospects of TME-targeted therapy [ 3, 4]. However, the current treatment of tumors typically merely focuses on tumor cells, and the TME is frequently overlooked or ignored in tumor therapy. This approach often leads to suboptimal treatment outcomes and potential recurrence of the tumor, as the complex interactions and influences within the TME are not taken into account. The disregard for the TME might also limit the development and efficacy of novel therapeutic modalities aimed at achieving more comprehensive and long-lasting tumor control.

The tumor microenvironment (TME) is constituted by numerous types of cells and extracellular matrix. As one of the most prevalent noncancerous cell types within the TME, cancer-associated fibroblasts (CAFs) have been thoroughly examined and are recognized to be implicated in a wide range of cellular processes, such as cell differentiation, proliferation, cell migration, and apoptosis. These processes play crucial roles in the development, progression, and metastasis of tumors, and the understanding of the functions and mechanisms of CAFs has become an important area of research in oncology [5]. The interaction between CAFs and other components of the TME also contributes to the complexity and heterogeneity of the tumor microenvironment, influencing the response to therapeutic interventions and prognosis of cancer patients. All of these can play critical roles in tumor biological behaviors, including tumorigenesis, tumor growth, angiogenesis, tumor progression, recurrence, and metastasis [ 5– 7]. It has been reported that the various components in TME can promote the conversion of normal fibroblasts (NFs) to CAFs in multiple ways [5]. However, the underlying mechanism of the interaction between breast cancer cells and fibroblasts, especially how breast cancer cells promote the transformation of NFs into CAFs, has not been fully investigated.

Exosomes are one subtype of membrane-contained vesicles with a diameter of about 40–100 nm that contain bioactive molecules, including microRNAs, DNAs, proteins, *etc*. [ 8 – 10]. They can be secreted by many types of cells to their surroundings and act as intercellular mediators between tumor cells and the TME. Exosomes discharged by tumor cells have been demonstrated to modify the TME, thereby facilitating tumor growth, immunosuppression, neovascularization, and metastatic advancement. MicroRNAs (miRNAs) are single-stranded small noncoding RNAs of about 20 nucleotides that can modulate the translation of target mRNAs and can regulate the TME by being internalized into exosomes and transferred into adjacent cells through exosomes [ 11– 13].

In the present study, we found that exosomal miR-105-5p from breast cancer cells could be internalized into fibroblasts and transform NFs into CAFs by downregulating LATS2, leading to malignant positive feedback regulation between breast cancer cells and CAFs. Furthermore, exosomes from highly metastatic breast cancer cells contained much more miR-105-5p than those from poorly metastatic breast cancer cells and thus were more efficient at transforming NFs into CAFs through LATS2/NF-κB signaling. In conclusion, this study highlights the important role of exosomal miR-105-5p from breast cancer cells in promoting the transformation of NFs to CAFs and suggests that miR-105-5p-LATS2 is a potential target for the treatment of breast cancer.

## Materials and Methods

### Cell culture and isolation of exosomes

Human breast epithelial cells (MCF-10A) and human breast cancer cells (MCF-7 and MDA-MB-231) were purchased from the American Type Culture Collection (ATCC, Manassas, USA). MCF-10A cells were cultured with mammary epithelial cell growth medium (Sigma-Aldrich, Merck KGaA, Darmstadt, Germany) containing 100 ng/mL cholera toxin (2 mg vials; C-8052; Sigma, Grand Island, USA) and 10% horse serum (GIBCO, Grand Island, USA). MCF-7 and MDA-MB-231 cells were cultured in DMEM (Sigma-Aldrich, Merck KGaA)/RPMI-1640 (Thermo Fisher Scientific, Waltham, USA) supplemented with 10% fetal bovine serum (FBS).

For extraction of exosomes, FBS was centrifuged for 70 min at 4°C and 110,000 *g* to eliminate exosomes, and MCF-10A, MCF-7 and MDA-MB-231 cells were cultured at 37°C with 5% CO _2_ in 10-cm plates with exosome-depleted complete medium for 2 days. Then, the exosomes in the conditioned medium (CM) were centrifuged from the supernatants at 120,000 *g* for 90 min and named Exo-MCF-10A, Exo-MCF-7 or Exo-MDA-MB-231. The size of the exosomes was detected via an electron microscope (FEI Tecnai G2 Spirit, Thermo Fisher Scientific) and Nanosight lm10 system (Nanosight, Navato, USA).

### Data analysis of exosomal miRNAs

The GEO database was used to query the expression of exosomal miRNAs in MDA-MB-231 and MCF-10A cells. The information was collected from the GSE50429 dataset, 31 miRNAs ( Supplementary Table S1) with significantly differential expression were identified, and the top 10 miRNAs were selected for further qRT-PCR verification.

### Extraction and culture of NFs

Normal fibroblasts were obtained from freshly resected paracancerous normal tissues (5 cm away from the edges of tumors) from breast cancer patients at the Affiliated Hospital of Shandong Second Medical University. Informed consent was obtained from each patient before surgery. The tissues were cut into pieces and cultured in complete culture medium. The spindle cells grew from the bottom of the tissue pieces 5–7 days later and were identified as fibroblasts [14]. These spindle NFs were harvested via enzymatic digestion and transferred to 48-well culture plates for expanded cell culture. Then, the NFs were divided into three groups and treated with Exo-MCF-10A, Exo-MCF-7 or Exo-MDA-MB-231 by supplementation of the corresponding exosomes into the culture media of the different NF groups.

### Knockdown and overexpression experiments

The LATS2 cDNA sequence was cloned and connected into the pcDNA3.1 vector to construct pcDNA-LATS2 overexpression plasmid. miR-105-5p mimics, inhibitors, si-LATS2, siRNA-MBNL1, siRNA-YTHDC1, siRNA-RBMY1A1 and corresponding control constructs were obtained from Genechem (Shanghai, China). Cells were cultured for 48 h before transfection. When the cells have grown to 70%–80% density of the six-well plate, we transfected the cells with plasmids encoding target sequences or siRNAs using Lipofectamine 3000 reagent (Invitrogen, Carlsbad, USA) according to the manufacturer’s instructions. After 48 h of transfection, RT-qPCR assay was used to detect the transfection efficiency and carry out follow-up experiments. Details of sequences used in this study were listed in Supplementary Table S2.

### F-actin measurement

F-actin content was detected as previously described [15]. Fibroblasts from the Exo-MCF-10A, Exo-MCF-7 and Exo-MDA-MB-231 groups were treated with or without TGF-β (10 ng/mL) stimulation. The cells were then fixed, permeabilized, washed, stained and measured via fluorescence analysis. The F-actin content was calculated by the following equation:

F-actin content = (F-actin *_t_* – F-actin _0_)/F-actin _0_

where, F-actin *_t_* is the fluorescence intensity at time *t*, and F-actin _0_ is the initial fluorescence intensity at time 0. All the experiments were repeated at least three times.

### Migration assay

For the migration assay, 1 × 10 ^5^ NFs were plated in the upper chambers of 24-well transwell plates (Corning Co., Corning, USA), and Exo-MCF-10A, Exo-MCF-7 or Exo-MDA-MB-231 was added. Five fields of migrated cells on the lower side of the membrane were randomly selected and counted under a microscope (200×) [16].

### RNA immunoprecipitation (RIP) analysis

Cells cultured in 10 cm plates were washed twice with ice cold PBS and scraped off in 1mL PBS. Then the cells was centrifuged and resuspended in an equal volume of intact RIP lysis buffer (Merck Millipore, Billerica, USA). Cell lysate were incubated overnight at 4°C with 100 pmol of synthetic single stranded biotinylated miR-105-5p or biotinylated mutant miR-105-5p oligonucleotides. Add agarose beads (Invitrogen) to each binding reaction and further incubate at 4°C for 4 h. Finally, wash the precipitate five times and boil it in SDS buffer for analysis by western blot analysis or RT-qPCR. IgG is used as a negative control.

### Western blot analysis

The cells or exosomes were dissolved in RIPA buffer and centrifuged at 10,000 *g* (4°C) for 20 min. The following antibodies were used in this study: LATS2 (ab243657, 1:1000; Abcam, Cambridge, USA), TSG101 (ab125011, 1:1000; Abcam), CD63 (ab134045, 1:1000; Abcam), CD9 (ab236630, 1:1000; Abcam), p65 (ab32536, 1:1000; Abcam), β-actin (ab8227, 1:1000; Abcam), IKB alpha (ab32518, 1:1000; Abcam), IKK alpha (ab32041, 1:1000; Abcam), FAP (E1V9V) (66562S, 1:1000; Cell Signaling Technology, Danvers, USA), p-AKT (Thr308) (4056S, 1:1000; Cell Signaling Technology), p-AKT (Ser473) (4060S, 1:2000; Cell Signaling Technology), AKT (pan) (4691S, 1:1000; Cell Signaling Technology), Cofilin (5175S, 1:1000; Cell Signaling Technology), p-Cofilin (Ser3) (3313S, 1:1000; Cell Signaling Technology), LIMK1 (3842S, 1:1000; Cell signaling technology), p-LIMK1 (Thr508) (3841S, 1:1000; Cell signaling technology) and HRP-linked Anti-rabbit IgG antibody (7074S, 1:2000; Cell signaling technology).

### Immunohistochemistry

Immunohistochemistry (IHC) was conducted using paraffin-embedded specimens of the mouse lung metastatic tissues. Briefly, post-deparaffinization, rehydration, and heat-induced antigen retrieval, specimens were incubated with rabbit anti-α-SMA (BF9212; Affinity Biosciences, Cincinnati, USA) and anti-FAP (52818; Cell Signaling Technology, Danvers, USA) specific antibodies overnight at 4°C. Specimens were then washed and incubated with anti-rabbit secondary antibodies (8114; Cell Signaling Technology), followed by exposure to DAB developer (P0203; Beyotime Biotech, Shanghai, China) and hematoxylin (MS4008; Maokangbio, Shanghai, China). Photos were taken under an optical microscope (Olympus, Tokyo, Japan).

### Collagen contraction assay

Briefly, 4 × 10 ^5^ NFs suspended in 500 μL of DMEM supplemented with 150 μL of Rat Tail collagen type I (3 mg/mL, 354236; BD, San Jose, USA) and 10 μL of 1 M NaOH were seeded in a 24-well plate and cultured for 18 h. ImageJ software was used to measure the gel area and evaluate contraction [17].

### RNA extraction and reverse transcriptase quantitative real-time PCR (qRT-PCR)

Total RNA from cells or exosomes was extracted via TRIzol reagent (Invitrogen) and reverse-transcribed into cDNA via the Prime Script RT Reagent Kit (Takara, Tokyo, Japan). The expression of mature miRNAs was detected via real-time quantitative PCR (qPCR) via SYBR Green Master Mix (Thermo Fisher Scientific) on an ABI 101 7500 real-time PCR system (Applied Biosystems, Foster City, USA). The data were evaluated via the 2 ^–ΔΔCt^ method and normalized to the level of the small nuclear RNA *U6* in advance. The sequences of primers are listed in Supplementary Table S3.

### Luciferase assay

The binding sites of miR-105-5p and *LATS2* were predicted via ENCORI. According to the prediction, wild-type and mutated control sequences, both containing luciferase reporter vectors (pGL3-Basic), were synthesized and named pGL3-LATS2 WT and pGL3-LATS2 MUT, respectively. pGL3-LATS2 WT and miR-105-5p mimics/empty vector or pGL3-LATS2 MUT and miR-105-5p mimics/empty vector were cotransfected into NFs via Lipofectamine 3000 (Invitrogen). miR-105-5p mimics (Genechem, Shanghai, China). The cell lysates were harvested 48 h after transfection, and firefly and Renilla luciferase activities were measured via a dual luciferase reporter assay kit (GenePharma, Shanghai, China) according to the manufacturer’s protocol.

### *In vivo* metastasis

NFs were pretreated with PBS, Exo-MCF-7, Exo-MDA-MB-231, or miR-NC and transfected with miR-105-5p. Then, MDA-MB-231/NF mixtures at a ratio of 1:5 (5 × 10 ^4^ MDA-MB-231 cells and 2.5 × 10 ^5^ differentially pretreated NFs, 100 μL) were inoculated into the third mammary gland pad of BALB/c nude mice (4 weeks old; Junke Bioengineering, Nanjing, China). The Institutional Animal Care and Use Committee (IACUC) approved the animal experiments, which were performed in accordance with the institution’s guidelines and principles for animal research. After 6 weeks, all the mice were sacrificed, and lung metastases were observed and assessed via histological and immunohistochemical examinations.

### Statistical analysis

All the experiments were repeated at least three times. Data analysis was performed via Microsoft Excel and GraphPad Prism 7. All the quantitative data are presented as the mean ± SD. The quantitative significance of differences between two groups was determined by Student’s *t* test unless otherwise stated. *P* < 0.05 was considered statistically significant.

## Results

### Exosomes from breast cancer cells promote the transformation of NFs to CAFs

**Figure 1:**
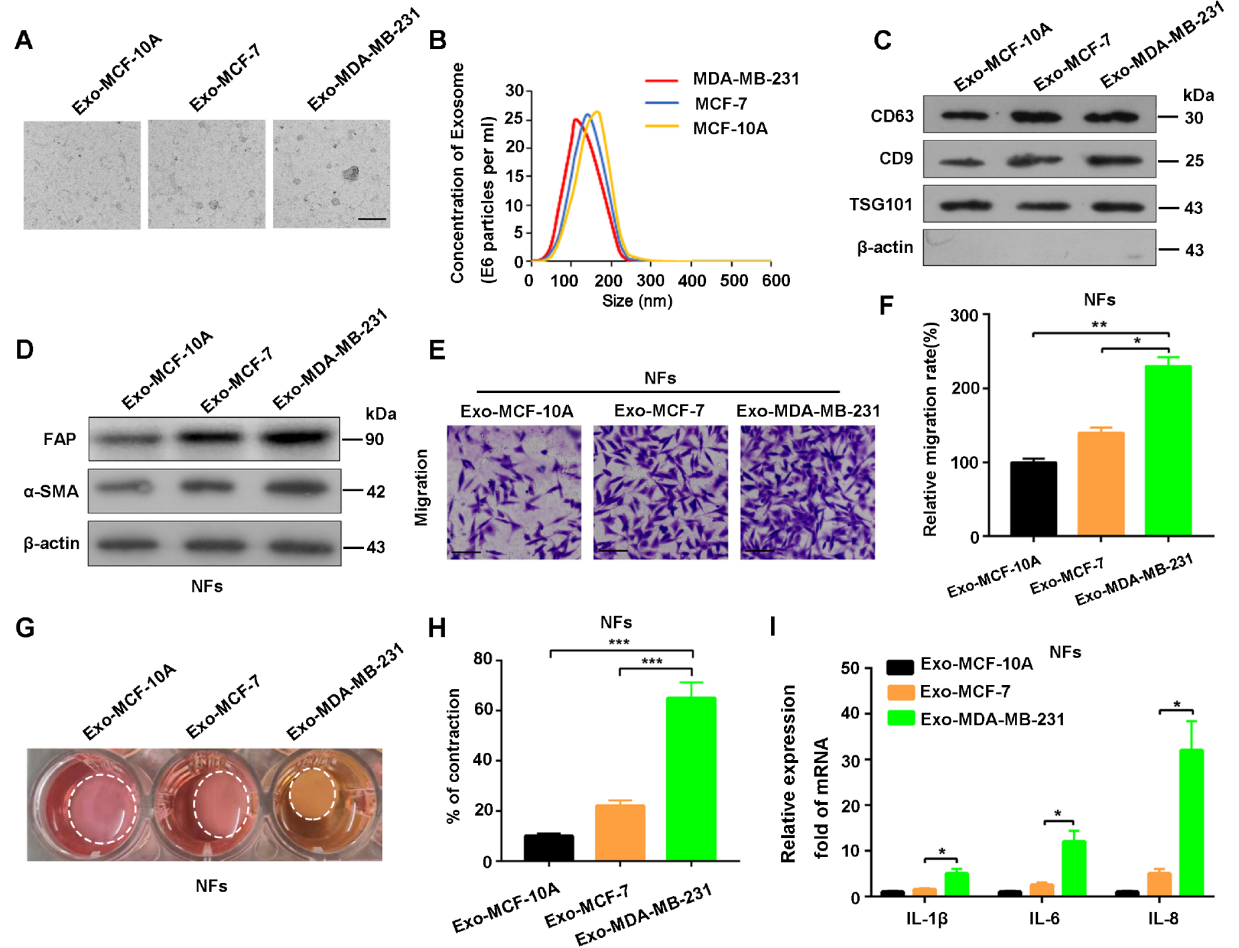
Figure 1 Breast cancer-derived exosomes mediate NF activation (A) TEM morphologies of exosomes extracted from MCF-10A, MCF-7 and MDA-MB-231 cells; scale bar = 0.2 μm. (B) Diameters analyzed by particle size analysis. (C) Exosomal biomarkers were tested by western blot analysis. (D) α-SMA and FAP expressions in NFs treated with Exo-MCF-10A, Exo-MCF-7 or Exo-MDA-MB-231 were analyzed by western blot analysis. (E) Representative images and (F) counting statistics of the Transwell assay results for NFs treated with Exo-MCF-10A, Exo-MCF-7 or Exo-MDA-MB-231. (G) Collagen contraction assay and (H) counting statistics of NFs treated with Exo-MCF-10A, Exo-MCF-7 or Exo-MDA-MB-231; representative images of three replicates at 96 h are shown. (I) qRT-PCR of the mRNA expressions of IL-1β, IL-6 and IL-8 in the three groups of NFs. *P < 0.05, **P < 0.01, ***P < 0.001. TEM, transmission electron microscopy; Exo-MCF-10A, Exo-MCF-7 and Exo-MDA-MB-231 are abbreviations for exosomes extracted from MCF-10A, MCF-7 and MDA-MB-231, respectively.

The normal breast epithelial cell line MCF-10A, the low-metastatic breast cancer cell line MCF-7 and the highly metastatic breast cancer cell line MDA-MB-231 were used to investigate the exosome-mediated interactions between breast cancer cells and fibroblasts. Exosomes from MCF-10A, MCF-7 and MDA-MB-231 cells were named Exo-MCF-10A, Exo-MCF-7, and Exo-MDA-MB-231 ( Figure 1A–C), respectively, and added to the culture medium of NFs. Interestingly, the breast cancer cell-derived exosomes Exo-MCF-7 and Exo-MDA-MB-231 induced increased expression of α-SMA and FAP in fibroblasts, and the potency of Exo-MDA-MB-231 was greater than that of Exo-MCF-7 ( Figure 1D). Moreover, the migration and contraction abilities of fibroblasts incubated with Exo-MDA-MB-231 were markedly greater than those of those incubated with Exo-MCF-7 ( Figure 1E–H). More importantly, fibroblasts educated with Exo-MDA-MB-231 produced higher levels of proinflammatory cytokines than those educated with Exo-MCF-7 ( Figure 1I). Collectively, these results suggest that exosomes from highly metastatic breast cancer cells are more potent at promoting the transformation of NFs to CAFs than are those from poorly metastatic breast cancer cells.

### Exosomal miR-105-5p mediated the transformation of NFs to CAFs

**Figure 2:**
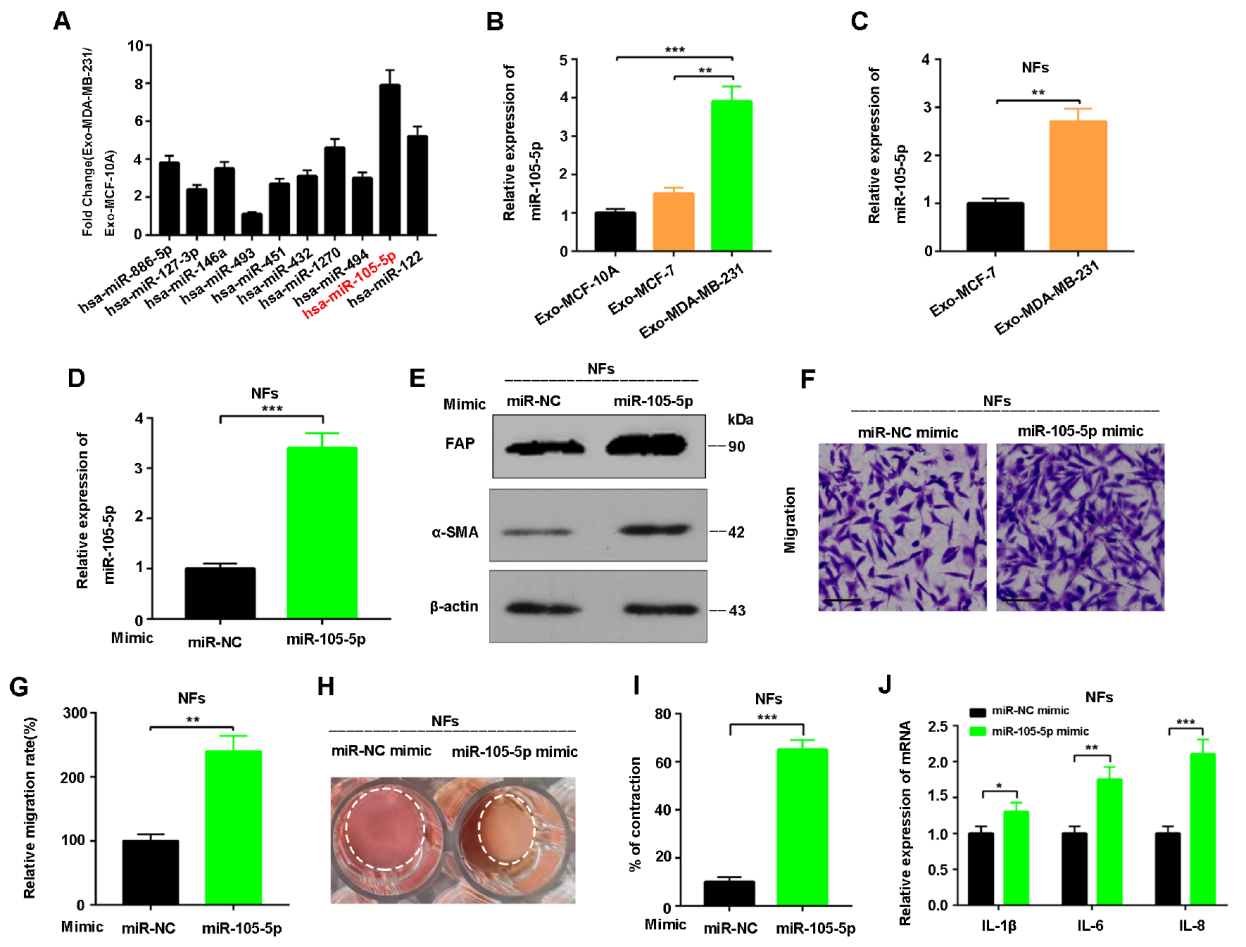
Figure 2 Exosomal miR-105-5p mediates NF activation (A) qRT-PCR of the expressions of the top 10 differential miRNAs between Exo-MDA-MB-231 and Exo-MCF-10A. (B) qRT-PCR of the expression of miR-105-5p in Exo-MDA-MB-231, Exo-MCF-10A and Exo-MCF-7 cells. (C) qRT-PCR of the expression of miR-105-5p in NFs treated with Exo-MDA-MB-231 or Exo-MCF-7. (D) qRT-PCR of the expression of miR-105-5p in NFs transfected with the miR-105-5p mimic or miR-NC. (E) α-SMA and FAP levels in NFs transfected with the miR-105-5p mimic or miR-NC were analyzed by western blot analysis. (F) Representative images and (G) counting statistics of the Transwell assay results for NFs transfected with the miR-105-5p mimic or miR-NC. (H) Representative contractility images of NFs transfected with the miR-105-5p mimic and (I) counting statistics (three replicates at 96 h). (J) qRT-PCR of the mRNA expressions of IL-1β, IL-6 and IL-8 in NFs transfected with the miR-105-5p mimic or miR-NC. *P < 0.05, **P < 0.01, *** P < 0.001.

Next, bioinformatics analysis of miRNAs in Exo-MCF-10A, Exo-MCF-7 and Exo-MDA-MB-231 was performed, and 31 upregulated miRNAs in Exo-MDA-MB-231 were identified ( Supplementary Table S1). The top ten miRNAs in Exo-MDA-MB-231 were detected via qRT-PCR, and the results revealed that the level of miR-105-5p was the highest ( Figure 2A). In addition, the miR-105-5p level in Exo-MDA-MB-231 was significantly greater than that in Exo-MCF-7 ( Figure 2B). Consistently, qRT-PCR analysis revealed more miR-105-5p in fibroblasts incubated with Exo-MDA-MB-231 than in those incubated with Exo-MCF-7, indicating that exosome-mediated sorting of miR-105-5p into fibroblasts occurred ( Figure 2C). Furthermore, exosomal miR-105-5p levels in clinical breast cancer samples were significantly greater than those in normal breast tissue samples ( Supplementary Figure S1A), while the survival rate of patients with higher miR-105-5p levels was obviously lower than that of patients with lower miR-105-5p levels ( *P* = 0.0016, Supplementary Figure S1B). Finally, the effect of miR-105-5p on the transformation of NFs into CAFs was verified via the transfection of miR-105-5p mimics/specific inhibitors into NFs ( Figure 2D–J and Supplementary Figure S2A–F). In conclusion, these findings revealed that exosomal miR-105-5p from breast cancer cells mediated the transformation of NFs to CAFs.

### The sorting of miR-105-5p into exosomes is mediated by RBMY1A1

**Figure 3:**
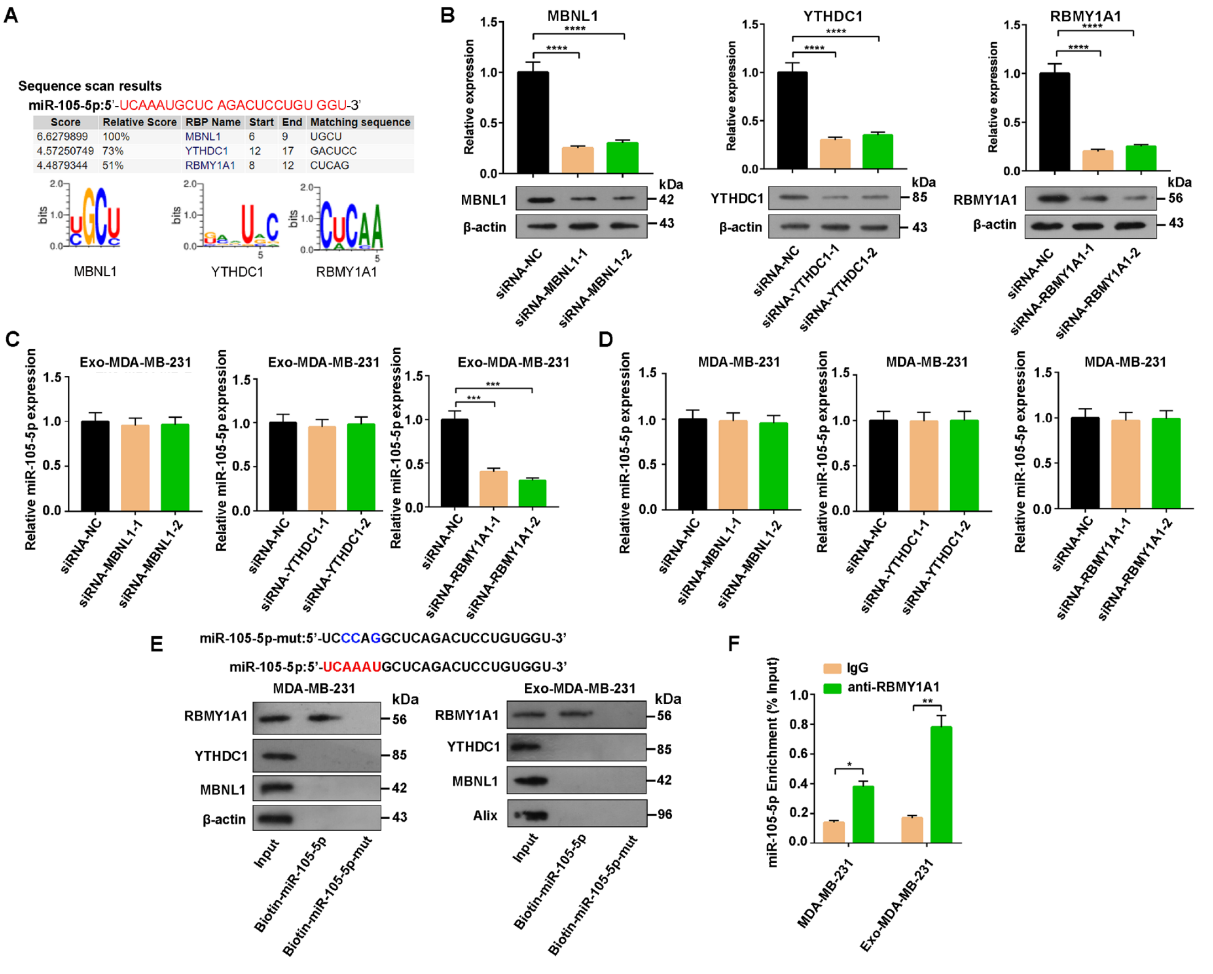
Figure 3 The sorting of miR-105-5p into the exosomes of breast cancer cells is mediated by RBMY1A1 (A) RBPDB analysis of the specific interaction between the miR-105-5p sequence and RBP motifs. (B) The expressions of MBNL1, YTHDC1 and RBMY1A1 in MDA-MB-231 cells transfected with the corresponding siRNAs (48 h later). (C) qRT-PCR of miR-105-5p expression in Exo-MDA-MB-231 after MBNL1, YTHDC1 and RBMY1A1 were silenced with the corresponding siRNAs. (D) qRT-PCR of miR-105-5p expression in MDA-MB-231 cells after RBMY1A1, MBNL1 and YTHDC1 was silenced respectively. (E) The expressions of MBNL1, YTHDC1 and RBMY1A1 in MDA-MB-231 cells and Exo-MDA-MB-231 after the transfection of biotin-labeled miR-105-5p. (F) RIP assays with anti-RBMY1A1 antibody or IgG were performed on the lysates of MDA-MB-231 cells or Exo-MDA-MB-231. *P < 0.05, **P < 0.01, ***P < 0.001, ****P < 0.0001.

To investigate whether miR-105-5p is specifically packaged into exosomes, the specific interaction between the miR-105-5p sequence and RNA binding protein (RBP) motif was analyzed via an RBP-specific database (RBPDB, http://rbpdb.ccbr.utoronto.ca). The results revealed that RBMY1A1, MBNL1 and YTHDCI have specific binding sites for miR-105-5p ( Figure 3A). Next, antisense RNAs were designed to target RBMY1A1, MBNL1 and YTHDC1 ( Figure 3B). Interestingly, only antisense RNA for RBMY1A1 markedly decreased exosomal miR-105-5p, whereas the other two antisense RNAs did not. Moreover, the cellular levels of miR-105-5p were not obviously affected by any of the antisense RNAs ( Figure 3C,D). These findings indicate that RBMY1A1 plays a crucial role in mediating the packaging of miR-105-5p into exosomes. Moreover, the binding of wild-type miR-105-5p and RBMY1A1 in MDA-MB-231 cells was detected by immunoprecipitation ( Figure 3E). Consistently, RIP analysis revealed that the RBMY1A1 antibody strongly enriched miR-105-5p, whereas the IgG antibody did not ( Figure 3F). Collectively, these data demonstrated that miR-105-5p was specifically packaged into exosomes by RBMY1A1.

### Exosomal miR-105-5p directly targets *LATS2* in fibroblasts

**Figure 4:**
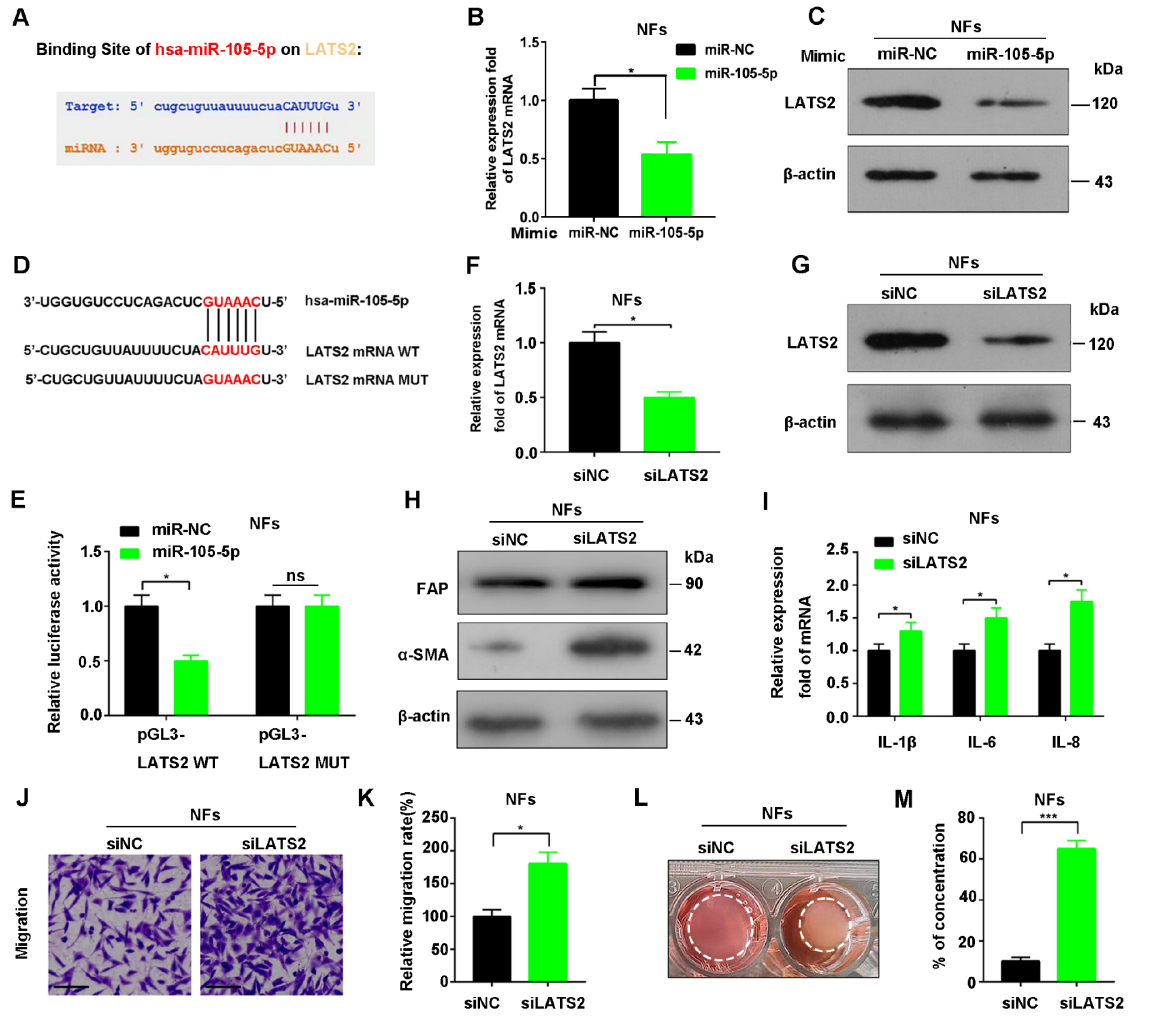
Figure 4 LATS2 is involved in the activation of NFs (A) Predicted target genes of miR-105-5p from ENCORI. (B) Influence of miR-105-5p on LATS2 mRNA expression detected by qRT-PCR. (C) The influence of miR-105-5p on LATS2 protein expression. (D) Binding site of miR-105-5p and LATS2. (E) Targeted binding of miR-105-5p to LATS2 revealed by luciferase assay. (F) LATS2 mRNA expression in NFs after transfection with siLATS2 or siNC. (G) LATS2 protein expression in NFs after transfection with siLATS2 or siNC. (H) α-SMA and FAP expressions in NFs transfected with siLATS2 or siNC were analyzed by western blot analysis. (I) qRT-PCR of the mRNA expressions of IL-1β, IL-6 and IL-8 in NFs transfected with siLATS2 or siNC. (J,K) Representative images (J) and quantification of NF migratory ability (K) after transfection with siLATS2 or siNC. Scale bar = 50 mm. (L,M) Representative images (L) and the degree of collagen contraction in NFs (M) detected via a collagen contraction assay after the transfection of siLATS2 or siNC (three replicates, 96 h). *P < 0.05, **P < 0.01, ***P < 0.001.

To investigate the functional contribution of exosomal miR-105-5p to the phenotypic switch of NFs, we identified the target of miR-105-5p in fibroblasts via ENCORI, and large tumor suppressor gene 2 ( *LATS2*) was found to be a direct target of miR-105-5p ( Figure 4A). qRT-PCR and western blot analysis confirmed that miR-105-5p inhibited *LATS2* mRNA and protein expressions ( Figure 4B,C), indicating that miR-105-5p regulates LATS2 at the translational level. To further verify the direct action of miR-105-5p on LATS2, the binding sites of miR-105-5p-LATS2 were predicted, and a luciferase reporter assay was performed with luciferase vectors containing wild-type LATS2 (LATS2-WT) and mutant LATS2 (LATS2-MUT) ( Figure 4D). The luciferase activity of LATS2-WT was significantly inhibited by miR-105-5p ( *P* < 0.05), whereas that of LATS2-MUT was not ( *P* > 0.05, Figure 4E), demonstrating that *LATS2* is the direct target of miR-105-5p. In addition, the expression of LATS2 in breast cancer tissues was significantly lower than that in normal breast tissues ( Supplementary Figure S3A). Similarly, the expression of LATS2 in breast cancer cells was also significantly lower than that in normal breast epithelial cells ( Supplementary Figure S3B). Furthermore, *LATS2* knockdown ( Figure 4F,G) markedly enhanced the expressions of α-SMA, FAP, IL-6, IL-8 and IL-1β ( Figure 4H,I), as well as the mobility and contraction of fibroblasts ( Figure 4J–M), whereas LATS2 overexpression reversed these effects ( Supplementary Figure S4A–F). In brief, these data suggested that LATS2 is a direct target of miR-105-5p and that the downregulation of LATS2 by miR-105-5p correlates with the transformation of NFs to CAFs.

### miR-105-5p activated fibroblasts via the LATS2-NF-κB signaling pathway

**Figure 5:**
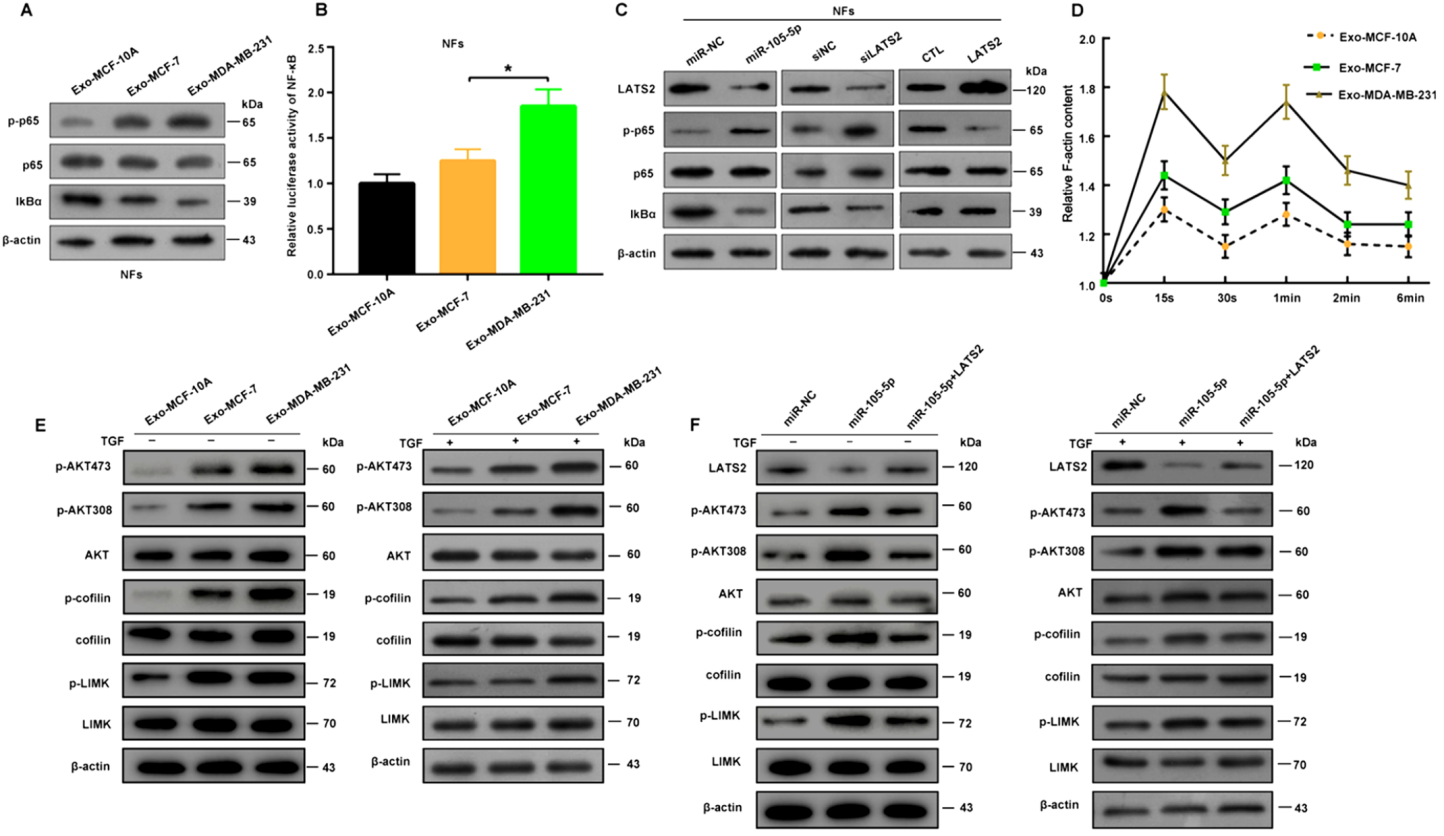
Figure 5 Exosomal miR-105-5p activates fibroblasts through the NF-κB pathway (A) p-p65, p65 and IκBα expressions in NFs treated with Exo-MCF-7 or Exo-MDA-MB-231 detected by western blot analysis. (B) Relative luciferase activity of NF-κB in NFs treated with Exo-MCF-7 or Exo-MDA-MB-231. (C) The expression of LATS2 and the activity of NF-κB signaling in NFs after overexpression of miR-105-5p or LATS2 or knockdown of LATS2. (D) Time course of the relative F-actin content in NFs treated with Exo-MCF-10A, Exo-MCF-7 or Exo-MDA-MB-231 (TGF-β, 10 ng/mL). The data are representative of at least three independent experiments. (E) Western blot analysis of the phosphorylation of AKT, cofilin and LIMK in total NF (10 ng/mL TGF-β stimulation for 30 min) lysates treated with Exo-MCF-10A, Exo-MCF-7 or Exo-MDA-MB-231, respectively; β-actin was used as a loading control. (F) Western blot analysis of the phosphorylation of AKT, cofilin and LIMK in total cell lysates from the miR-NC, miR-105-5p and miR-105 + LATS2 groups after stimulation with 10 ng/mL TGF-β for 30 min; β-actin was used as a loading control. *P < 0.05.

Given that the NF-κB signaling pathway is involved in fibroblast activation [16] and that LATS2 is a negative regulator of NF-κB signaling [18], we wondered whether NF-κB could be modulated by miR-105-5p-LATS2. As shown in Figure 5A,B, Exo-MDA-MB-231 activated NF-κB signaling in fibroblasts by promoting the phosphorylation of p65 and decreasing IκBα expression, whereas Exo-MCF-7 had much lower efficacy in activating NF-κB signaling. Furthermore, in accordance with the effect of Exo-MDA-MB-231 on NF-κB signaling, overexpression of miR-105-5p in NFs increased the activation of NF-κB signaling, whereas LATS2 overexpression in NFs inhibited NF-κB signaling activation ( Figure 5C).

Moreover, Exo-MDA-MB-231 can facilitate TGF-β-induced actin polymerization [19] and the phosphorylation of LIMK, cofilin and AKT [20] in fibroblasts ( Figure 5D,E). Consistently, the overexpression of miR-105-5p in fibroblasts can activate AKT/LIMK/cofilin signaling, whereas this activation can be reversed by LATS2 overexpression ( Figure 5F). Taken together, these findings indicate that exosomal miR-105-5p-LATS2 can promote fibroblast activation and CAF phenotype transformation by activating NF-κB signaling.

### Positive feedback between CAF transformation and breast cancer progression

**Figure 6:**
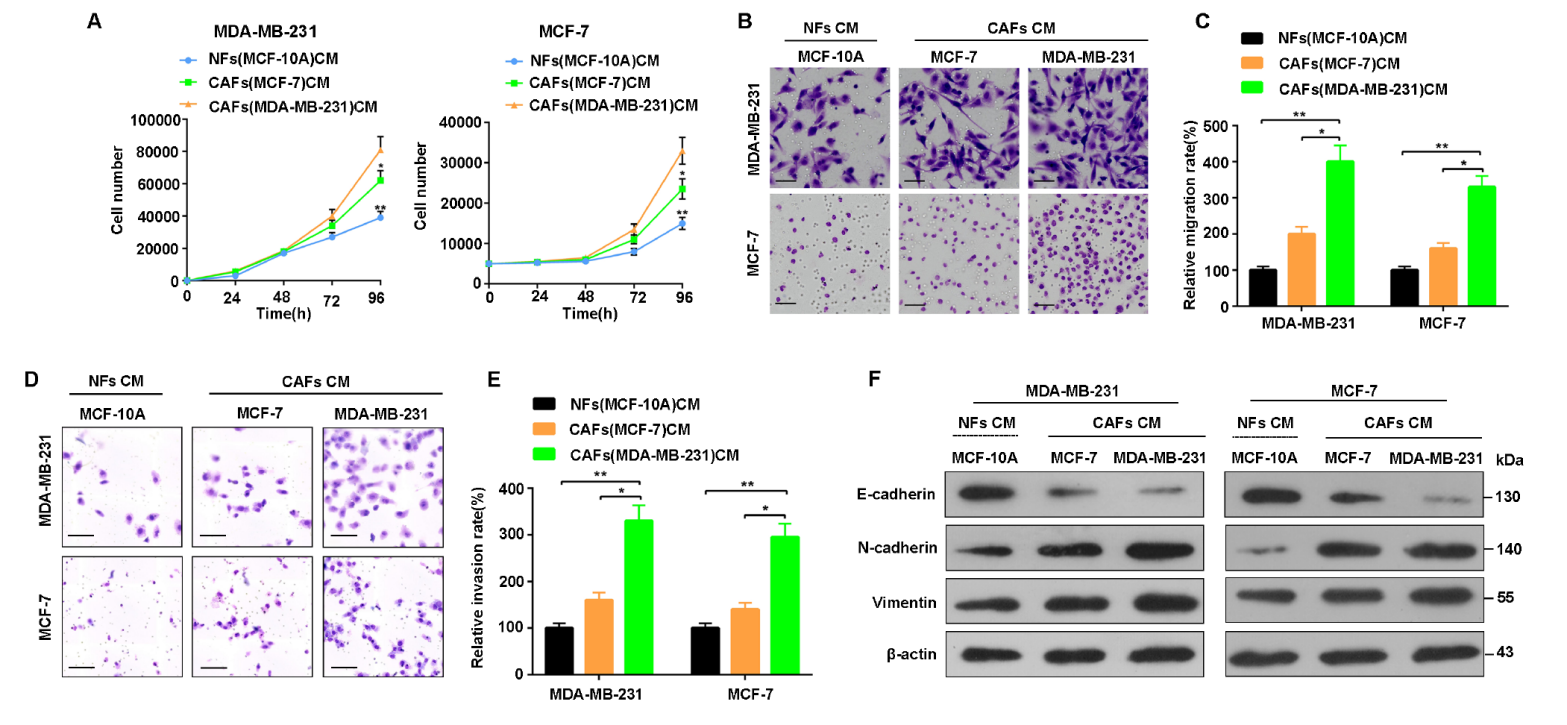
Figure 6 Activated fibroblasts promote the proliferation, invasion and EMT of breast cancer cells (A) Proliferation ability of MDA-MB-231 and MCF-7 cells detected by CCK8 assay. (B–E) Representative images and statistical analysis of the migration and invasion ability of MDA-MB-231 and MCF-7 cells detected by Transwell assay. (F) EMT markers in MDA-MB-231 and MCF-7 cells were detected by western blot analysis. *P < 0.05, **P < 0.01.

As mentioned above, exosomes derived from breast cancer cells promote the transformation of NFs to CAFs. On the other hand, conditioned medium of CAF (CAF-CM) was used to treat MDA-MB-231 and MCF-7 cells to investigate whether CAFs could accelerate breast cancer progression. The results showed that CAF-CM increased the proliferation, migration and invasion of MDA-MB-231 and MCF-7 cells ( Figure 6A–E). In addition, CAF-CM promoted EMT in breast cancer cells by increasing vimentin and N-cadherin expression and decreasing E-cadherin expression ( Figure 6F). These results suggest that CAFs promote the malignant characteristics of breast cancer cells and that there is a positive feedback loop between CAF transformation and breast cancer progression.

### Exosomal miR-105-5p facilitates the lung metastasis of breast cancer by inducing the transformation of NFs to CAFs *in vivo*

**Figure 7:**
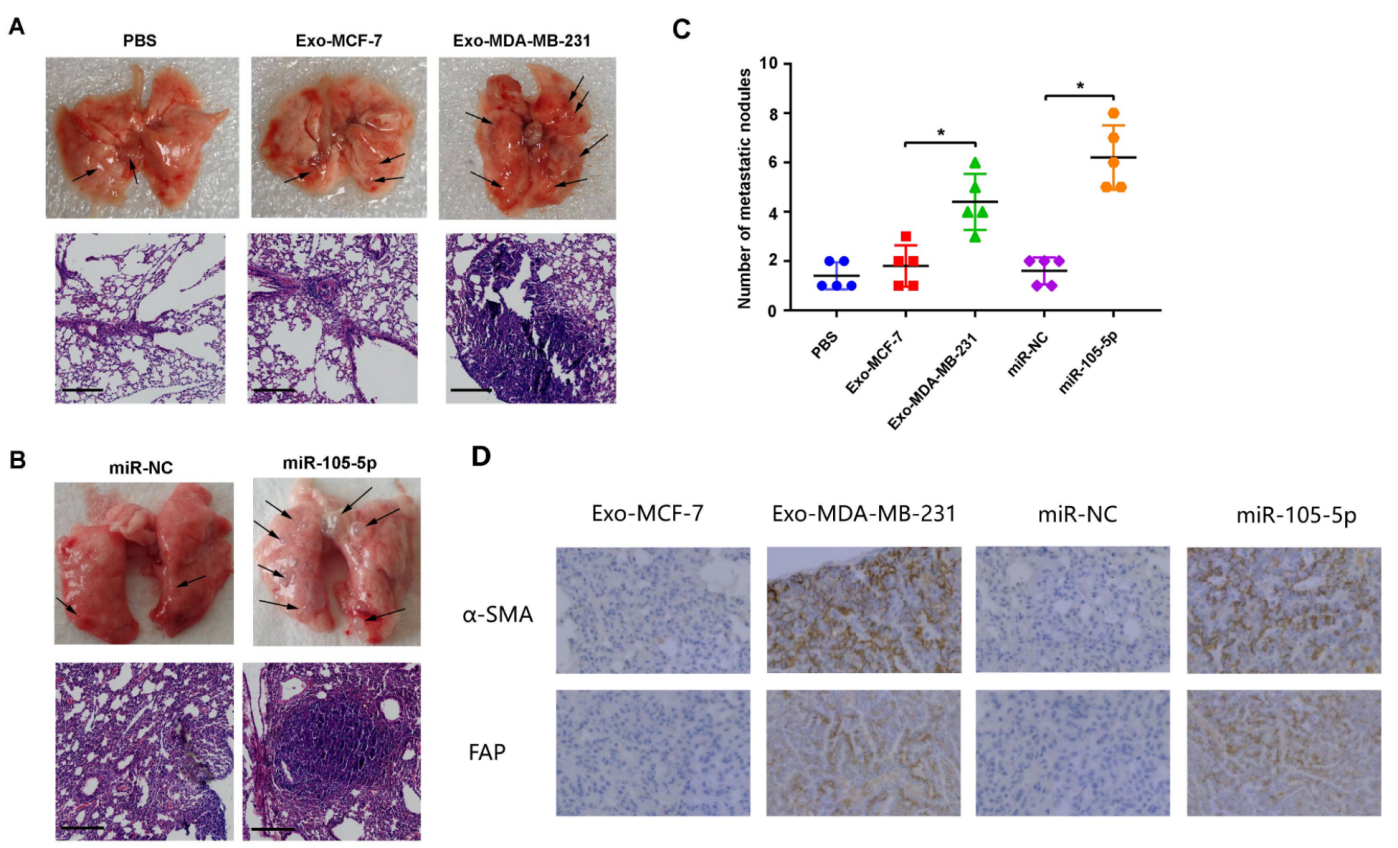
Figure 7 Exosomal miR-105-5p promotes breast cancer lung metastasis (A) Macroscopic and microscopic observations of mouse lung metastasis of mixed breast cancer cell NFs stimulated with Exo-MCF-7 or Exo-MDA-MB-231. (B) Macroscopic and microscopic observation of mouse lung metastasis of breast cancer cells transfected with miR-105-5p. (C) Number of mouse lung metastatic nodules per 100× magnification field. (D) Protein expressions of α-SMA and FAP in metastatic nodules were determined via IHC. Scale bar = 100 μm. *P < 0.05.

Finally, an *in vivo* experiment was employed to further verify the function of exosomal miR-105-5p in transforming NFs into CAFs. NFs were pretreated with PBS or subjected to Exo-MCF-7, Exo-MDA-MB-231, miR-NC or miR-105-5p transfection. MDA-MB-231 cells and differentially pretreated NFs were subsequently inoculated orthotopically into BALB/c mice. Six weeks later, all the mice were sacrificed, and lung metastases were observed. Both Exo-MCF-7 and Exo-MDA-MB-231 clearly increased the number of lung metastatic nodules, whereas significantly more metastatic nodules were observed in the Exo-MDA-MB-231 group ( Figure 7A,B). In addition, there were notably more metastatic nodules in the mice in the miR-105-5p group than in those in the miR-NC group ( Figure 7B,C). Furthermore, the expressions of α-SMA and FAP in the metastatic nodules of the Exo-MDA-MB-231 and miR-105-5p groups were obviously greater than those in the Exo-MCF-7 and miR-NC groups ( Figure 7D). These results indicated that exosomes from highly metastatic breast cancer cells, Exo-MDA-MB-231, can facilitate lung metastasis of breast cancer *in vivo* by inducing the transformation of NFs to CAFs via exosomal miR-105-5p.

## Discussion

The tumor microenvironment represents the site where tumor cells interact with stromal cells, which contributes to cancer malignancy. CAFs have been identified as critical regulators of tumor progression, and exosome-based crosstalk between tumor cells and CAFs occurs continuously in the TME. This study revealed that exosomes from breast cancer cells can be endocytosed into fibroblasts and mediate the transformation of NFs to CAFs. Moreover, high-metastatic breast cancer-derived exosomes were more likely to transform NFs into CAFs than low-metastatic breast cancer-derived exosomes. Moreover, Exo-MDA-MB-231 significantly enhanced the inflammatory secretion of activated NFs, which in turn was involved in positive feedback to the malignant progression of breast cancer cells.

To date, exosomes are considered intercellular molecular shuttles packaged with RNA, protein, DNA and microRNA that can be transferred into neighboring cells to modulate their physiological and pathological processes [21]. Exosome-based miRNA delivery is one of the approaches used by cancer cells to influence adjacent components in the TME [22]. In this study, bioinformatics analysis identified miR-105-5p as the most differentially expressed miRNA in Exo-MDA-MB-231, and exosomal miR-105-5p could be internalized into NFs and induce CAF transformation, which led to epigenetic changes such as increased α-SMA and FAP expression, increased migration and contraction ability, and increased secretion of inflammatory factors. Consistent with corresponding reports on miR-105-5p [ 23– 25], these findings further revealed novel functions of exosomal miR-105-5p.

As a downstream kinase of the Hippo pathway, *LATS2* is a tumor suppressor-modulating gene, and downregulation of LATS2 can promote the progression of different types of cancers [ 18, 26] . Here, our data showed that exosomal miR-105-5p could specifically target the 3′UTR of *LATS2* mRNA and repress its expression at the translational level. Nuclear factor-κB (NF-κB) has been found to be closely related to exosome-mediated cancer progression [ 27– 30], and this research further demonstrated that miR-105-5p-LATS2 significantly activates the NF-κB pathway, which is involved in NF-CAF transformation.

In conclusion, our findings suggest that in breast cancer cells, miR-105-5p can be specifically internalized into exosomes and that exosomal miR-105-5p can be taken up by NFs and then promote NF-CAF transformation by inhibiting LATS2 and activating NF-κB signaling. Moreover, exosomal miR-105-5p-induced NF-CAF transformation contributes to a positive feedback loop between CAFs and breast cancer cells, which accelerates the malignant progression of breast cancer. This study provides new insight into the interplay between tumor cells and fibroblasts, suggesting a potential therapeutic strategy for breast cancer.

## Supplementary Data

Supplementary data is available at *Acta Biochimica et Biophysica Sinica* online.
